# Impact of viral drift on vaccination dynamics and patterns of seasonal influenza

**DOI:** 10.1186/1471-2334-13-589

**Published:** 2013-12-13

**Authors:** Yanyu Xiao, Seyed M Moghadas

**Affiliations:** 1 Agent-Based Modelling Laboratory, York University, Toronto, Ontario, M3J 1P3, Canada

## Abstract

**Background:**

Much research has been devoted to the determination of optimal vaccination strategies for seasonal influenza epidemics. However, less attention has been paid to whether this optimization can be achieved within the context of viral drift.

**Methods:**

We developed a mathematical model that links different intra-seasonal dynamics of vaccination and infection to investigate the effect of viral drift on optimal vaccination for minimizing the total number of infections. The model was computationally implemented using a seasonal force of infection, with estimated parameters from the published literature.

**Results:**

Simulation results show that the pattern of large seasonal epidemics is strongly correlated with the duration of specific cross-protection immunity induced by natural infection. Considering a random vaccination, our simulations suggest that the effect of vaccination on epidemic patterns is largely influenced by the duration of protection induced by strain-specific vaccination. We found that the protection efficacy (i.e., reduction of susceptibility to infection) of vaccine is a parameter that could influence these patterns, particularly when the duration of vaccine-induced cross-protection is lengthened.

**Conclusions:**

Given the uncertainty in the timing and nature of antigenically drifted variants, the findings highlight the difficulty in determining optimal vaccination dynamics for seasonal epidemics. Our study suggests that the short- and long-term impacts of vaccination on seasonal epidemics should be evaluated within the context of population-pathogen landscape for influenza evolution.

## Background

The presence of host immunity is essential for the generation and maintenance of population protection, referred to as ‘herd immunity’ [1]. This immunity can be influenced by natural infection, vaccination, and the immunological status of individuals in the population. In the epidemiological context, waning immunity (post infection or vaccination) can lead to vastly different outcomes compared to the lack of ‘pathogen-specific immunity’ (in the absence of prior exposure or vaccination) [2]. For slow-mutating pathogens (i.e., timelines for their evolution is longer than the average life-span of the host population), waning immunity can be parameterized in epidemic models to represent an increased susceptibility of the hosts [3]. However, for fast-mutating pathogens (e.g., influenza), both waning and the lack of pre-existing immunity play important roles in determining disease dynamics [1,2]. For these types of infection, prior immunity caused by exposure to, or vaccination against, predecessor strains may not confer protective functional activity against newly emerged strains of the same pathogen [2,4,5].

The concept of herd immunity has two important implications: (i) theoretically, it means that the vaccine need not be 100% effective; (ii) practically, not every susceptible individual needs to be vaccinated, implying that a vaccination coverage (fraction of susceptible individuals to be vaccinated) below 100% may suffice for epidemic control [6]. However, the level of herd immunity is affected by several key parameters governing the transmission dynamics, including the duration of vaccine-induced immunity that wanes over time; pathogen evolution that can lead to antigenically distant variants for which pre-existing immunity has little or no protective effects [7,8]; and the circulation of pathogen strains, which decelerates the decline of herd immunity by boosting the host immune-level through re-exposure [1,8]. These could influence both short- and long-term epidemiological outcomes of vaccination, and may lead to unintended consequences (e.g., generation of immune-escape variants) [9], as a result of changes in the patterns of evolutionary responses and the fitness landscape of the pathogen [10]. This underscores the importance of considering transmission dynamics and pathogen evolution simultaneously in order to formulate effective vaccination strategies [11].

Despite the existence of a large body of literature on vaccination against seasonal influenza epidemics, optimizing the impact of vaccine-induced protection remains elusive [12]. This is partly due to the abovementioned factors, which influence herd immunity, rendering its effect too short-lived for any lasting epidemiological impact. However, previous work has highlighted the importance of three interrelated mechanisms that portray the landscape for host-pathogen interactions, namely disease evolution, invasion, and prevention [13]. In this study, we made a systematic attempt to include the effect of these mechanisms in a population dynamical model to link the dynamics of disease transmission within an influenza season to the epidemiological patterns between seasonal epidemics. Our objectives were to: (i) illustrate how vaccine-related parameters (i.e., protection efficacy and duration of vaccine-induced protection) influence the dynamics of infection in a season; and (ii) determine the effect of vaccine distribution on changing the patterns of epidemics between distinct seasons caused by immunologically-related strains. While the conceptual modelling framework relies on a simple deterministic model of susceptible-infected-recovered (SIR) structure, we applied pulse theory for the inclusion of seasonal vaccination [14].

## Methods

To include the effect of genetic drift on transmission dynamics and prevention in our model, we considered two main factors: (i) the gradual reduction in effective protection of pre-existing immunity conferred by vaccination against, or natural infection caused by, a similar genetic subtype of one influenza strain (and this reduction is largely caused by viral drift which lessens the neutralizing effect of pre-existing antibody-mediated immunity); and (ii) the duration of pre-existing immunity (and this corresponds to genetic distance of successor variants which depends on frequency and strength of viral drift between seasonal epidemics).

We included vaccine efficacy in the model as a parameter that reduces the susceptibility of vaccinated individuals to acquire infection during the upcoming season for which vaccination is administered. We assumed that the duration of cross-protective immunity resulted from vaccination (and before it becomes fully ineffective), is less or equal to that conferred by natural infection. This also confers partial protection following vaccination or exposure to infection with gradual decrease in its effectiveness against successor strains. During the partial protection era, if individuals are vaccinated or exposed to an immunologically related strain, their level of protective immunity is boosted, thereby reducing their susceptibility for a longer period of time. However, without vaccination or re-exposure, the reduction in protective levels of pre-existing immunity will result in a continuous increase in susceptibility to infection.

### Model structure and assumptions

To develop a population dynamical model, we divided the population into four main compartments comprising of susceptible, vaccinated, infected, and recovered individuals. For the dynamics of a seasonal epidemic (with a relatively short duration), we excluded demographic factors, such as birth and natural death. We assumed that the buildup immunity with recovery from infection will prevent re-infection with the circulating strain in the same season. The immunity against circulating strains of influenza in each season is generated through natural infection during the season or vaccination before the start of season. We assumed that immunity induced by infection or vaccination in each season will provide partial protection against circulating strains in the subsequent influenza seasons, and this (cross-protection) immunity becomes gradually less effective due to the continual drift of influenza viruses [1]. We also assumed an imperfect vaccine-induced protection, and therefore vaccinated individuals may become infected with an average transmission rate that is lower than that of susceptible individuals. Furthermore, as the level of (cross-protection) immunity conferred by natural infection or vaccination decreases over time, the corresponding risk of acquiring infection increases. While re-infection does not occur in the same season, recovered individuals from previous seasons or previously vaccinated individuals can acquire infection with a transmission rate, which depends on the level of cross-protection at the time of exposure, and is a function of age since previous infection or vaccination. The level of immunity generated by natural infection is assumed to be higher, with cross-protection effects that last longer than vaccination.

**Figure 1:**
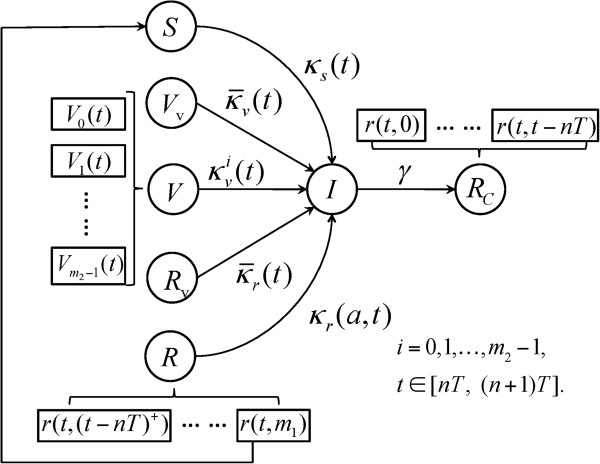
**The transitions between model compartments for season *n*.** In this model, *S* (*t*) is the number of fully susceptible individuals at the beginning of the current season; *V*_0_ (*t*) is number of individuals who received vaccines for the current season; *V*_*i*_ (*t*) is the number of individuals whose last vaccination was given *i* seasons ago; *V*_*v*_ (*t*) is the number of previously vaccinated individuals who also received vaccination for the current season; *r*(*t, α*) is the number of individuals who were recovered from infection at time *α* (0 < *α < m*_1_); *R* (*t*) is the total number of recovered individuals at time *t*; *R*_*v*_ (*t*) is the number of previously recovered individuals who received vaccination for the current season; *I* (*t*) is the number of infections at time *t* during the current season; and γ is the rate of recovery from infection.

**Figure 2:**
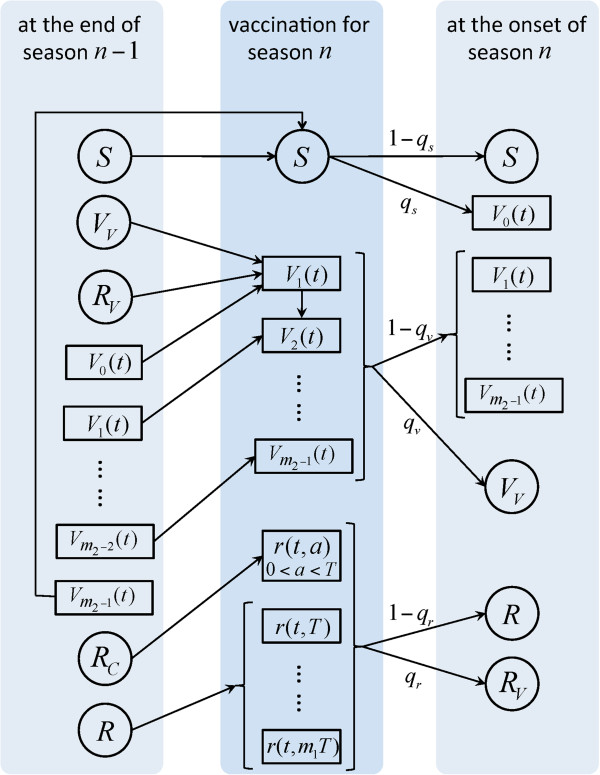
The structure of between-season model with vaccination.

Given the normal practice for vaccination, we assumed that vaccines are deployed before the start of a seasonal epidemic. Vaccination was assumed to reduce susceptibility to infection by generating some level of immunity in vaccinated individuals. The duration and protection efficacy of naturally-acquired and vaccine-induced immunity for subsequent seasonal epidemics were varied as key parameters in the model simulations. We developed the epidemic model formulated by a system of impulsive delay differential equations with age structures (i.e., age since last infection or vaccination) to capture a pulse-like vaccination strategy. We assumed that all vaccines are distributed prior to the start of each season (see Additional file 1). The dynamics of within and between seasonal epidemics are schematically represented in Figures 1 and 2.

### Within-season dynamics

For the dynamics of a seasonal epidemic, we divided the total population into five compartments (See Figure 1): susceptible individuals (*S*), previously vaccinated individuals (*V*_*i*_, *i* = 1, 2,.., *m*_2_ - 1), newly vaccinated individuals for the upcoming season (*V*_0_, *V*_*v*_, *R*_*v*_), infected individuals (*I*), and recovered (*R*, *R*_*c*_) individuals. We further classified newly vaccinated individuals based on their prior status as being part of a susceptible, vaccinated, or recovered class in the previous season. For those from a recovered class, we considered two subgroups of individuals, consisting of recovery from infection in the current season (*R*_*c*_) and recovery from infection in a previous season (*R*). Except for individuals who recovered during the current season (with no possibility of re-infection), all the other groups can become infected through contacts with infectious individuals. During each season, those who are in the recovery group and whose age post-recovery reaches *m*_1_, where *m*_1_ represents the maximum time that cross-protection from natural infection lasts, will become fully susceptible and move to the *S* compartment.

The transmission rates for different compartments depend on time (for seasonality) and age since the last infection or vaccination. The infection dynamics are governed by a seasonally-force *P*_*j*_(*t*), j = *s,v,r* where *s,v,r* correspond to the classes of susceptible, vaccinated, and recovered individuals. The corresponding baselines of transmission rates are given by $\beta_{s},\beta_{v}^{i}\left(t\right),{\bar{\beta }}_{v},\beta_{r}\left(a\right)$ and ${\bar{\beta }}_{r}$, where *i* is the number of seasons following the last vaccination, and varies from 1 to *m*_2_ - 1. Since the level of immunity will be further boosted by vaccination at the beginning of each season for previously recovered or vaccinated individuals, the baselines transmission rates for those compartments are reduced and expressed by:

$$\begin{array}{l} \;{\bar{\beta }}_{v}=\beta_{v}^{0}\left(0\right)\cdot (1-\sigma ),\\ \;{\bar{\beta }}_{r}=\beta_{r}\left(0\right)\cdot (1-\sigma ), \end{array}$$

where *σ* represents the vaccine efficacy against infection. The relations between *β*_*r*_(*a*), $\beta_{v}^{i}\left(t\right)$, and *β*_*s*_ is discussed in Additional file 1, where *α* represents the age since last infection. For seasonality of influenza epidemics [15], we considered *P*_*j*_(*t*) = 1 + *ϵ*_*j*_ cos(2*πt*/*T*), where *T* is the period of seasonality (i.e., 1 year in our model), and *ϵ*_*j*_ is the amplitude of seasonal fluctuation. We defined the corresponding seasonal transmission coefficient for each class by *κ*_*s*_(*t*), $\kappa_{v}^{i}\left(t\right)$, ${\bar{\kappa }}_{v}\left(t\right)$, *κ*_*r*_(*t*, *a*), ${\bar{\kappa }}_{r}\left(t\right)$ as given in Table 1. The within-season dynamics of infection is schematically presented in Figure 1.

**Table 1:** **Description of model parameters and their associated values and ranges [**[15]**-**[17]**]**

**Parameter**	**Description**	**Value (range)**
*q*_*s*_,*q*_*v*_,*q*_*r*_	Fraction of individuals receiving vaccination	0 - 1
*σ*	Vaccine efficacy	0.6 – 0.95
*μ*	Fraction of population vaccinated	Variable
*m*_1_	Number of seasons that the protection acquired from natural infection lasts	2 - 8 seasons
*m*_2_	Number of seasons that the protection acquired from vaccination lasts	2 - 4 seasons
*γ*	Recovery rate from infection	0.2 day ^-1^
*ϵ*_*j*_	Amplitude of seasonal fluctuation	0.85
*α*	Age since last infection	Variable
$\beta_{s},\beta_{v}^{i}\left(t\right),\beta_{r}\left(a\right)$ *i* = 0, 1, ⋯, *m*_2_ - 1	Baseline transmission rates of infection for susceptible, vaccinated, and recovered individuals, respectively	*β*_*s*_ = 150/*N* year ^-1^ (*N*: total population size)
${\bar{\beta }}_{v}\left(t\right),{\bar{\beta }}_{r}$	Baseline transmission rates of infection for previously vaccinated and recovered individuals, respectively, who also received vaccine for the current season	Variable
*κ*_*s*_(*t*) = *β*_*s*_ · *P*_*s*_(*t*)	Transmission rate of infection for susceptible class	Variable
$\kappa_{v}^{0}\left(t\right)=\beta_{v}^{0}\left(t\right)P_{s}\left(t\right)$	Transmission rate of infection for newly vaccinated from susceptible class	Variable
$\kappa_{v}^{i}\left(t\right)=\beta_{v}^{i}\left(t\right)P_{v}\left(t\right)$	Transmission rate of infection for previously vaccinated individuals who received their last vaccine *i* seasons ago	Variable
*i* = 1, 2, ⋯, *m*_2_ - 1		
${\bar{\kappa }}_{v}\left(t\right)={\bar{\beta }}_{v}\cdot P_{v}\left(t\right)$	Transmission rate of infection for previously vaccinated individuals who also received vaccine for the current season	Variable
*κ*_*r*_(*t*, *a*) = *β*_*r*_(*a*) · *P*_*r*_(*t*)	Transmission rate of infection for recovered individuals with the recovery age α since last infection	Variable
${\bar{\kappa }}_{r}\left(t\right)={\bar{\beta }}_{r}\cdot P_{r}\left(t\right)$	Transmission rate of infection for recovered individuals who received vaccine for the current season	Variable

### Between-season dynamics

For the dynamics of between seasons, we regroup the compartments of within-season dynamics to three main classes: susceptible (*S*), vaccinated (*V*), and recovered (*R*) individuals. Before the current season starts, a fraction of individuals in each of these classes are vaccinated with a total number of *q*_*s*_*S* + *q*_*v*_*V* + *q*_*r*_*R* = *μN* vaccine doses distributed, where *N* is the total population size (and therefore *μ* represents the fraction of population vaccinated), and *q*_*s*_, *q*_*v*_, *q*_*r*_ are the fraction of individuals vaccinated in the corresponding classes. After vaccination, we considered the dynamics of within-season with the corresponding classes as defined in the previous section. The schematic diagram for the model of between-seasons is presented in Figure 2, and further details of the model structure and system equations are provided in Additional file 1.

### Parameterization and initial conditions

We parameterized the model with estimated values from published literature [15-17]. Several parameters were varied in simulations to identify possible changes to the dynamics of between-seasons. Parameter values and their associated ranges are summarized in Table 1. The initial conditions used in our simulations are: *S*(0) = 10^6^, *I*(0) = 1, *V*_*v*_(0) = 0, *R*_*v*_(0) = 0, *V*_*i*_(0) = 0, *i* = 1, 2, …, *m*_2_ - 1, *r*(0, *a*) = 0.

### Computational implementation

We implemented the model using C*++* in Matlab to perform simulations. The impulsive delay differential equations were solved numerically using forward-time central-space algorithm and midpoint method using four recursive steps described in Additional file 1. At the beginning of each simulation run, we assigned initial values to different population compartments. Based on these initial conditions, *q*_*s*_ and *q*_*v*_ were varied in the feasible region given by *Ω* = {(*q*_*s*_, *q*_*v*_)| 0 ≤ *q*_*s*_, *q*_*v*_ ≤ 1, *q*_*s*_*S*^0^ + *q*_*v*_*V*^0^ ≤ *μN*} to seed the simulations for season *n*, where *S*^0^ and *V*^0^ are the total numbers of susceptible and previously vaccinated individuals before the start of vaccination for season *n*. Depending on the values of *S*^0^ and *V*^0^, and the size of vaccine stockpile, *q*_*s*_ and *q*_*v*_ may be less than 1. For each pair of (*q*_*s*_, *q*_*v*_) in Ω, the model governed by the integro-differential equations system (see Additional file 1) was simulated to determine the final size of epidemic (i.e., total number of infections throughout the epidemic) in each season. For each season, we discretized the parameter space Ω, and stored data of the final sizes for all population compartments to proceed with the random or optimal selection of the initial conditions for the next season, given all possible pairs of (*q*_*s*_, *q*_*v*_).

For the random vaccination, a pair of (*q*_*s*_, *q*_*v*_) was randomly selected in the current Ω space, and the associated simulation results were adopted as the initial conditions for the next season. For the optimal selection, we searched the entire parameter space Ω to determine pairs of (*q*_*s*_, *q*_*v*_) that minimized the epidemic final size (for the current season *n*), given by

$$J_{\text{min}}^{n}=\underset{\left(q_{s},q_{v}\right)\in \Omega }{\text{min}}\;\int_{\mathrm{nT}}^{\left(n+1\right)T}\mathrm{\gamma I}\left(\eta \right)\;\mathrm{d\eta}.$$

The simulated populations at the end of each season were used as the initial conditions for the next season. In each scenario, *q*_*r*_ was determined using the relation *q*_*s*_*S* + *q*_*v*_*V* + *q*_*r*_*R* = *μN*.

## Results

We considered a susceptible population of 10^6^ individuals for season 1, and seeded simulations with an infected individual in each season. We chose *μ* = 0.1, 0.2 corresponding to 10% and 20% vaccination coverage of the total population. Simulations were run for vaccine efficacies of *σ* = 0.6, 0.8 (corresponding to an average of 60% and 80% reduction in susceptibility to infection post vaccination [16,17]) with different durations of cross-protective immunity induced by natural infection (*m*_1_) and vaccination (*m*_2_) [15,18].

### Random vaccine distribution

**Figure 3:**
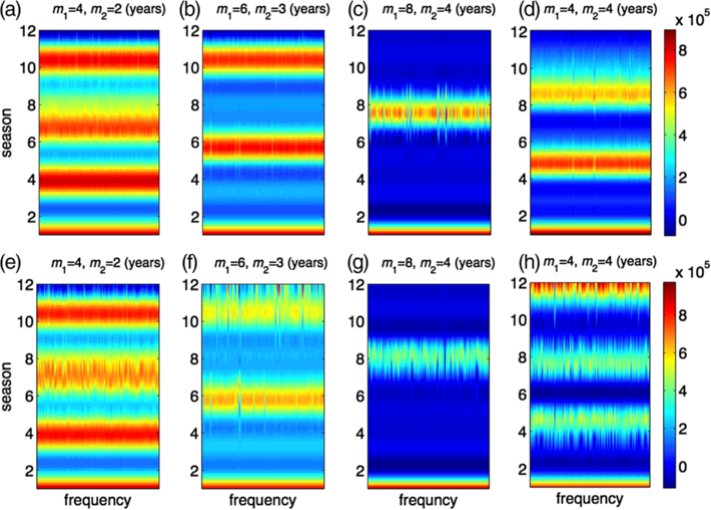
**Patterns of seasonal epidemics for 12 seasons with 100 independent simulation runs when vaccines are randomly distributed.** Colorbars represent the total number of infections for each season; and *m*_1_ and *m*_2_ represent the durations of immunity induced by natural infection and vaccination, respectively, in the absence of re-exposure or vaccination following priming. Vaccine efficacy in prevention of infection is 80%, and the total fraction of the population vaccinated is 10% **(a,b,c,d)** and 20% **(e,f,g,h)**. Other parameter values are given in Table 1.

For each randomly selected pair of (*q*_*s*_, *q*_*v*_), we ran 100 independent simulations to explore the effect of duration of vaccine-induced and natural immunity on the seasonal patterns of epidemics. Figure 3a-d illustrate the patterns of epidemic final size over 12 seasons for different combinations of (*m*_1,_*m*_2_), when the vaccine efficacy is *σ =* 0.8 and the vaccine coverage is maintained at 10% of the total population. These simulations indicate that episodes of high epidemic sizes generally follow a pattern that corresponds to the duration of immunity induced by natural infection. As the duration of vaccine-induced immunity increases, lower epidemic sizes of subsequent seasons follow a high epidemic episode. We observed similar patterns with lower epidemic sizes when the vaccine coverage was increased to 20% of the total population (Figure 3e-h). Considering the randomness in vaccine distribution, these simulations suggest that the effect of vaccination on seasonal patterns of an epidemic is largely influenced by the genetic similarity of successor variants resulting from viral drift, which determines the lasting effects of natural immunity generated through exposure to predecessor strains.

**Figure 4:**
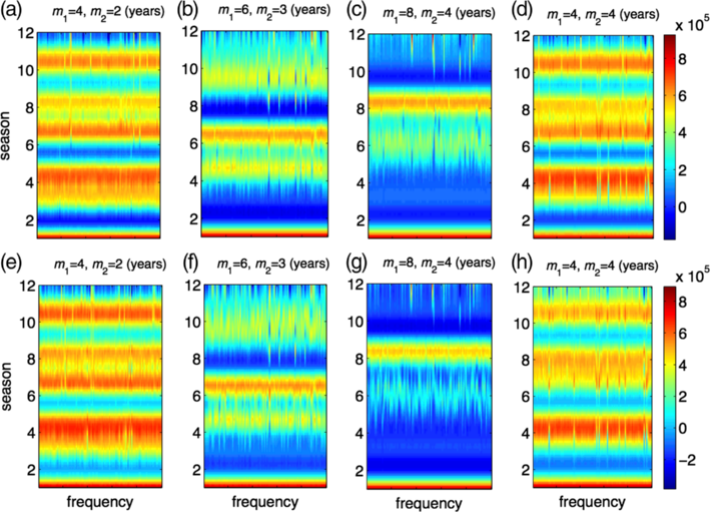
**Patterns of seasonal epidemics for 12 seasons with 100 independent simulation runs when vaccines are randomly distributed.** Colorbars represent the total number of infections for each season; and *m*_1_ and *m*_2_ represent the durations of immunity induced by natural infection and vaccination, respectively, in the absence of re-exposure or vaccination following priming. Vaccine efficacy in prevention of infection is 60%, and the total fraction of the population vaccinated is 10% **(a,b,c,d)** and 20% **(e,f,g,h)**. Other parameter values are given in Table 1.

**Figure 5:**
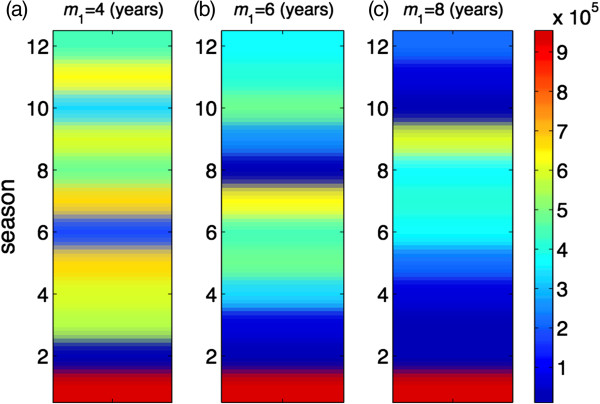
**Patterns of seasonal epidemics for 12 seasons in the absence of vaccination.** Colorbar represents the total number of infections for each season, and *m*_1_ is the duration of immunity induced by natural infection. Duration of immunity induced by natural infection is **(a)***m*_1_ = 4; **(b)***m*_1_ = 6; and **(c)***m*_1_ = 8 years. Other parameter values are given in Table 1.

In order to determine the effect of vaccine efficacy on seasonal patterns, we also simulated the model for similar scenarios when *σ =* 0.6. Simulation results, presented in Figure 4, demonstrate that the protection efficacy of vaccine is a parameter that could influence seasonal patterns, and lead to a large variation in epidemic sizes, particularly when the duration of vaccine-induced immunity increases. However, vaccine coverage has little impact on changing the seasonal patterns regardless of the lasting protection of vaccination. We also simulated the model in the absence of vaccination. As illustrated in Figure 5, not only do the patterns of seasonal epidemics differ, but also the final size of infections in each season could be substantially different (but not necessarily higher) compared to the corresponding scenarios in the presence of vaccination. These patterns are affected by the lack of vaccine-induced immunity, higher incidence of infections in some seasons, and potentially a higher level of herd immunity due to booster conferred by re-exposure to natural infection.

### Optimal vaccine distribution

**Figure 6:**
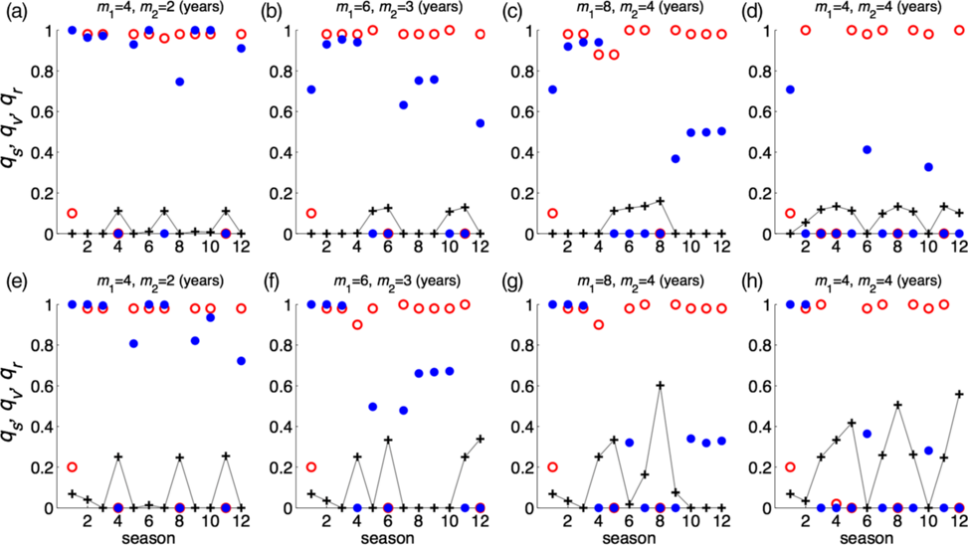
**Optimal vaccine distribution for 12 seasons to achieve the minimum number of infections in each season.** Vaccine efficacy in prevention of infection is 80%, and *m*_1_ and *m*_2_ represent the durations of immunity induced by natural infection and vaccination, respectively, in the absence of re-exposure or vaccination following priming. For each season, red circle, blue dot, and plus sign represent a possible scenario for vaccine distribution to susceptible, previously vaccinated, and previously recovered classes of individuals, respectively. The total fraction of the population vaccinated is 10% **(a,b,c,d)** and 20% **(e,f,g,h)**. Other parameter values are given in Table 1.

**Figure 7:**
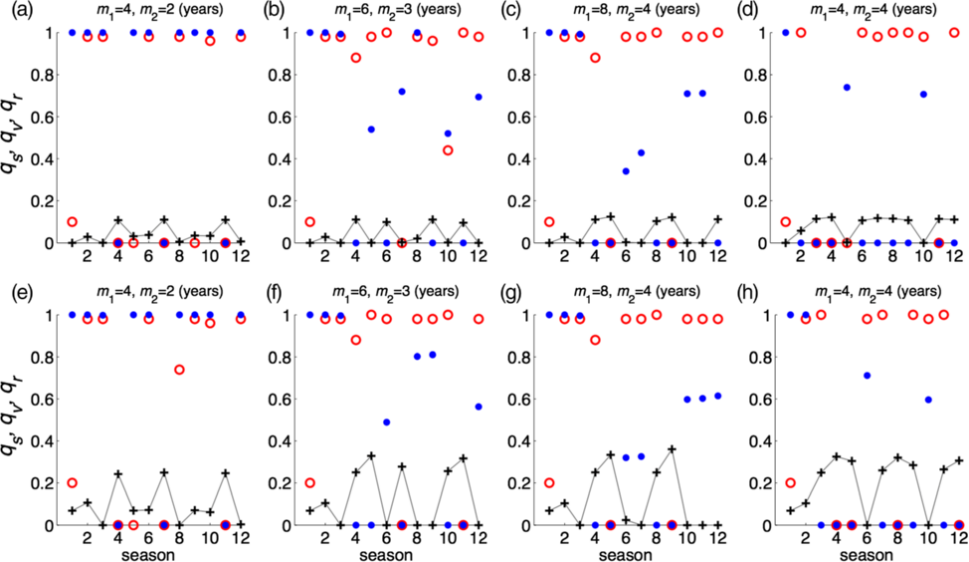
**Optimal vaccine distribution for 12 seasons to achieve the minimum number of infections in each season.** Vaccine efficacy in prevention of infection is 60%, and *m*_1_ and *m*_2_ represent the durations of immunity induced by natural infection and vaccination, respectively, in the absence of re-exposure or vaccination following priming. For each season, red circle, blue dot, and plus sign represent a possible scenario for vaccine distribution to susceptible, previously vaccinated, and previously recovered classes of individuals, respectively. The total fraction of the population vaccinated is 10% **(a,b,c,d)** and 20% **(e,f,g,h)**. Other parameter values are given in Table 1.

**Figure 8:**
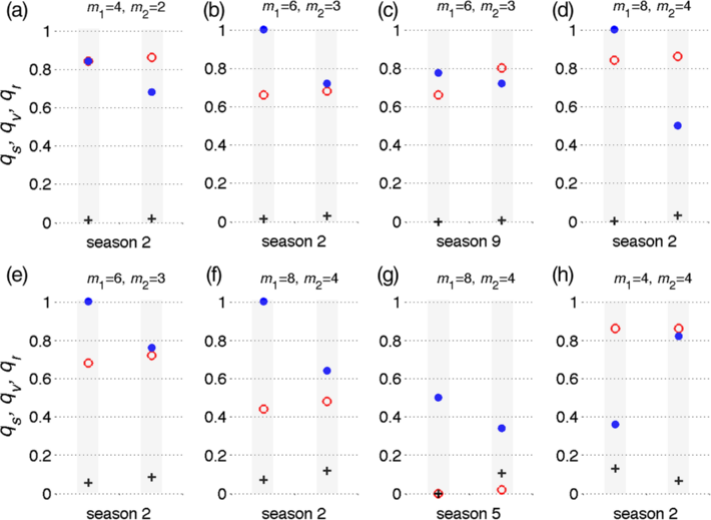
**Possible vaccine distributions to achieve the minimum number of infections in some seasons.** Vaccine efficacy in prevention of infection is 80%, and *m*_1_ and *m*_2_ represent the durations of immunity induced by natural infection and vaccination, respectively, in the absence of re-exposure or vaccination following priming. For simulated seasons, two columns include red circles, blue dots, and plus signs representing optimal scenarios for vaccine distribution to susceptible, previously vaccinated, and previously recovered classes of individuals, respectively. The total fraction of the population vaccinated is 10% **(a,b,c,d)** and 20% **(e,f,g,h)**. Other parameter values are given in Table 1.

For the optimal vaccine distribution, a global minimization search was applied to determine pairs of (*q*_*s*_, *q*_*v*_) in Ω for each season, where vaccination leads to the minimum number of infections throughout the season. Figure 6 shows vaccine distributions in 12 seasons where the vaccine efficacy was fixed at *σ =* 0.8. We observed no specific patterns for the optimal vaccine distribution, which is affected by the duration of cross-protection immunity induced by vaccine and natural infection. In most seasons, the optimal vaccine distribution corresponds to a high (or full) coverage of susceptible individuals regardless of the fraction of population vaccinated. When vaccine efficacy is lower (*σ =* 0.6), similar results were obtained for the optimal vaccine distribution (Figure 7a-h). Our simulations indicate that the minimum final size of the epidemic could be achieved with different pairs of (*q*_*s*_, *q*_*v*_) in Ω, suggesting that the optimal vaccine coverage of different subpopulations may not be unique. Figure 8 shows two possible scenarios for optimal vaccine distribution for different seasons with the vaccine efficacy of *σ =* 0.8. This further demonstrates the complexity of vaccination dynamics, even when the nature of antigenic drift is well predicted.

It is worth noting that the optimal vaccination fractions (*q*_*s*_, *q*_*v*_, *q*_*r*_) were determined based on the total number of vaccines (i.e., 10% or 20% of the total population size) available for each season. Having a large fraction of a subpopulation vaccinated in an optimal scenario does not necessarily correspond to a large number of vaccinated individuals. For example, *q*_*s*_ = 1 corresponds to 100% vaccination of the susceptible population; however, this indicates that the total number of susceptible individuals (with no cross-protection) for that particular season is lower than the total number of vaccines. Similarly, high coverage of vaccine for previously vaccinated or recovered individuals shows that their population sizes are less than the total number of vaccines. In contrast, a low coverage of vaccine in an optimal scenario could correspond to a low or relatively high subpopulation sizes.

## Discussion

Frequent generation of influenza virus mutations is an important drawback for disease control, as reflected in the isolation of vaccine-escape viral mutants and in the antigenic variation of viral populations [19]. The latter occurs at widely different rates due to the rapid drift of influenza viruses [14,20]. If the new variant strains generated by drift are significantly different from predecessor strains, cross-protection conferred by vaccination or natural infection may diminish, thereby enabling antigenically drifted viruses to effectively escape from herd immunity [21]. This effect appears to be a particular impediment to optimizing vaccination strategies for reducing the transmission and adaptation rates of new variants in the host population.

This study shows the effect of viral drift on vaccination dynamics by including two tracking paths in a dynamic model of seasonal influenza epidemics. First, continual viral drift lessens the strength of host immunity by reducing the effect of antibody titers for prevention of infection. This is irrespective of the functionality of the cellular immunity in clearance of infection and reduction in the severity of illness. Second, the rate of drift and its antigenic characteristics can enhance fitness and adaptation of the virus variants to a point that can cause the full escape of adaptive immune responses, thereby diminishing the level of herd immunity. Although adaptive immunity is a self-protection mechanism, its protective effects often extend well beyond the individual [22], since the existence of such immunity greatly influences the transmission dynamics of the pathogen in the population as a whole.

Our simulations show that the rise and fall of herd immunity by vaccination or natural infection can greatly influence epidemic patterns. These patterns could be further affected by the vaccine efficacy and duration of strain-specific immunity that may provide partial protection against drifted viruses. The results indicate that optimizing vaccine distribution for susceptible, previously vaccinated, and previously infected individuals, is confounded by several factors, most notably by the effect of viral drift on the pre-existing immune protection and its duration of partial functionality. The findings suggest that determining optimal vaccination strategies for seasonal influenza in the presence of viral drift is a challenging task, and may not be achievable given the uncertainty and variability in the level of pre-existing immunity in the population. Complicating matters further is the unpredictability of patterns of sequence diversity within seasons and the prevalence level of antigenically drifted viruses in different seasons [23].

Our study has several limitations that come from simplifying assumptions and the structure of the model. We considered a homogeneously mixing population for the incidence of infection. Realistically, population interactions are heterogeneous with complex networks due to the variability in population demographic characteristics, social patterns, behavioural responses, and movements [24,25]. These heterogeneities have been recognized as factors that could have immoderate effects on disease transmission and vaccination dynamics during an emerging pathogen [26]. Extending our model structure to a network or agent-based framework would enable the incorporation of these factors at levels that are computationally tractable [23,27]. Our model is based on the assumption of an even distribution of vaccines in different population compartments. Unevenness in the coverage of vaccines in different age groups of the population can be considered by developing an age-structure model, which will also allow for the consideration of different levels of vaccine efficacy that may be influenced by age, health status, and risk factors of individuals. While we have not measured age-based outcomes in our model, it has been recognized that attack rates of influenza infection among young age groups (e.g., school children) could be considerably higher than adults, due in part to their large number of contacts [28]. Vaccine prioritization of children has been suggested as a preventive measure that its effects could indirectly maximize population wide-benefits of immunization programs [29].

In the context of public health, optimization of vaccination translates to prioritization of different age-groups with varying degree of risk factors for infection and disease outcomes. However, immune-pathogen factors discussed in this study would still play an essential role in the presence of age-dependent dynamics. Furthermore, we assumed that the efficacy of vaccines remained constant through simulated seasons for each scenario. However, depending on the dominant strain of drifted viruses, vaccine efficacy may be different from one season to another. We also assumed a fixed duration of immunity induced by vaccination or natural infection in each simulated scenario for seasonal epidemics. However, such duration will depend on boosting of immunity through re-exposure to infection while having some level of pre-existing immunity, and the antigenic drift, which cannot be deterministically studied [30]. Despite these limitations, our study indicates that the viral drift has a profound impact on the optimal vaccination and epidemic dynamics, suggesting that the effect of vaccination should be considered within the context of population-pathogen landscape for influenza evolution.

## Conclusions

Although vaccination is a key preventive measure to reduce the burden of seasonal influenza epidemics, identification of optimal vaccine distribution remains a challenging task. Viral drift and duration of cross-reactive immunity (induced by vaccination or natural infection) appear to play a substantial role in vaccination dynamics during seasonal epidemics. Determining optimal vaccination strategies may not be achievable due to the unpredictability of the nature of viral drift and unknown protection levels of pre-existing immunity in the population. Our findings suggest that the effect of vaccination strategies should be evaluated within the context of evolutionary patterns of influenza viruses.

## Competing interests

The authors declare that they have no competing interests.

## Authors’ contributions

Designed the study: SM. Developed the model structure and simulation program: YX. Analyzed the data and simulation results: YX, SM. Wrote the first draft of the manuscript: SM, YX. Both authors contributed to the revisions and final version, read the paper, and approved it.

## Pre-publication history

The pre-publication history for this paper can be accessed here:

http://www.biomedcentral.com/1471-2334/13/589/prepub

## Supplementary Material

Additional file 1 Supplementary information. Click here for file
