# Supplementary material for: Impact of viral drift on vaccination dynamics and patterns of seasonal influenza
Source: BMC Infect Dis. 2013 Dec 13;13:589. doi: 10.1186/1471-2334-13-589 (PMC4028866; doi:10.1186/1471-2334-13-589)
Supplement: Additional file 1 — Supplementary information. [file 1471-2334-13-589-S1.pdf]

Supplementary Information

Impact of viral drift on vaccination dynamics and patterns of seasonal influenza

Yanyu Xiao, Seyed M. Moghadas

Agent-Based Modelling Laboratory, York University,

Toronto, Ontario, M3J 1P3 Canada

This supplementary text provides details of the model structure for both within- and between-season dynamics, with derivation of the full model using the concept of pulse vaccination [6].

1 Disease dynamics within a season

We assumed that the cross-protective immunity acquired through natural infection declines gradually in the absence of re-exposure to similar strains or vaccination [1]. We considered this decline in the model, and assumed that the pre-existing immunity (resulted from natural infection) becomes ineffective after m_1 seasons (due to continual viral drift). Consistent with the observed data, we considered the recurrence of seasonal epidemics with a period of $T = 1$ year. Let a denote the recovery age (the time-period elapsed since last infection), and $r(t,\, a) \in \mathbb{C}\,(\mathbb{R}^+ \times [0,\, m_1 T) \to \mathbb{R})$ represent the density of the individuals with the recovery age a at time t, as shown in Figure 1. Similar to the work of Metz and Diekmann [3], the dynamics of the density $r(t, a)$ at nth season

can be described by the following partial differential equation:

$$\Big(\frac{\partial}{\partial t}+\frac{\partial}{\partial a}\Big)r(t,a)=-\kappa_r(t,a)r(t,a)I(t),\quad t\in[nT,\,(n+1)T]. \tag{1.1}$$

where $\kappa_r(t,a)$ is the disease transmission rate for individuals in the recovery class. Here, we assume that no re-infection occurs within the same season due to the high level of immunity acquired from infection with the same pathogen. Incorporating the seasonal impact on disease transmission, one can write $\kappa_r(t,a), t\in[nT,(n+1)T)$ in the form of a step function,

$$\kappa_r(t,a)=\begin{cases}0 & 0\le a\le t-nT,\\ \beta_r(a)P_r(t) & t-nT<a<m_1T,\end{cases}$$

where $\beta_r(a)$ is an increasing function of age a since last infection (considering the increase in susceptibility to infection due to the reduced efficiency of pre-existing immunity); and $P_r(t)$ is a periodic function of time t reflecting seasonal variations. In this work, we adopted the two functions proposed in [5] for $\beta_r(a)$ and $P_r(t)$,

$$\beta_r(a)=\frac{\beta_s}{1+a_1e^{b_1/(a-m_1T)}},\quad a\in[0,m_1T)$$

and

$$P_r(t)=1+\epsilon_r\cos(2\pi t/T),$$

where β_s is the baseline transmission rate in the absence of any pre-existing immunity (i.e., fully susceptible); and a_1, b_1 and ϵ_r are constant coefficients. Furthermore, we assumed that the immune protection developed following natural infection, regardless of seasonality, is equivalent to that generated by vaccination: $\beta_r(0)=\beta_s(1-\sigma)$, where σ is the vaccine efficacy.

From the definition of $r(t,\,a)$, the total population of recovered individuals at time t can be expressed by

$$R(t)=\int_0^{m_1T}r(t,a)\,\mathrm{d}a. \tag{1.2}$$

The population of individuals with the recovery age $a=0$ is the number of individuals who have just recovered from infection. Thus,

$$r(t,0)=\gamma I(t). \tag{1.3}$$

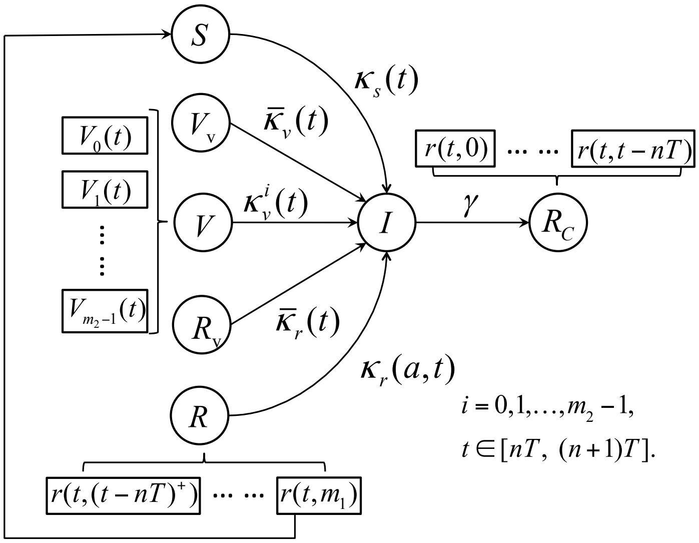

Figure 1: Model diagram for transitions between different population compartments within season n, where $n = 1, 2, \cdots$.

Individuals recovered m_1T seasons ago ($r(t, m_1T)$) will return to the susceptible class due to the loss of protective effects of pre-existing immunity.

Based on the practice of vaccination against influenza, we assumed that vaccination takes place between seasons, at time $t = 0, T, 2T, \cdots$, and considered it as a pulse vaccination strategy [4, 6] represented in Figure 2. Assuming that the protective effects of vaccine-induced immunity last for m_2 seasons after vaccination, we have m_2 compartments for vaccinated individuals with different levels of immunity. These include:

1) $V_0(t)$: the total number of susceptible individuals who have taken their most recent vaccination at the start of the current season;

2) $V_i(t)$, $i = 1, \cdots, m_2 - 1$: the total number of individuals for whom the most recent vaccination was received at i seasons ago.

Therefore, instead of having a continuous age structure (partial differential equation), we may

represent a (age post recent vaccination) by $t - nT + iT$, and use a discrete age structure to describe the loss of vaccine-induced protection during the nth season ($t \in [nT, (n+1)T)$) by the following ordinary differential equation,

$$\frac{\mathrm{d}V_i(t)}{\mathrm{d}t} = -\kappa_v^i(t)I(t)V_i(t), \qquad i = 0, 1, \cdots, m_2 - 1, \quad t \in [nT, (n+1)T),$$

where $\kappa_v^i(t) = \beta_v^i(t)P_v(t)$ is the disease transmission rate for V_i; $\beta_v^i(t)$ is an increasing function of $t - nT + iT$ corresponding to the reduction in the vaccine-induced protection; and $P_v(t)$ is a periodic function, reflecting the seasonality of disease transmission. We also assumed that $\beta_v^i(t)$ has a similar form as $\beta_r(a)$, i.e.,

$$\beta_v^i(t) = \frac{\beta_s}{1 + a_2 e^{b_2/(t-nT+iT-m_2T)}}, \qquad i = 0, 1, \cdots, m_2 - 1, \quad t \in [nT, (n+1)T)$$

where a_2 and b_2 are constant coefficients. Here, we set the transmission rate for newly vaccinated individuals as $\beta_v^0(0) = \beta_s(1 - \sigma)$, with σ representing the efficacy of vaccine (i.e., reduction in transmissibility due to vaccine-induced protection).

For compartments of individuals previously recovered within m_1 seasons or vaccinated within m_2 seasons (in what follows, we will write as 'previously recovered' or 'previously vaccinated'), and received vaccination at the beginning of the current season, we use the notation V_v and R_v, respectively.

2 Disease dynamics between seasons

At the end of season n (before the start of vaccination for season $n + 1$), those who still remained in the V_i class ($i = 0, 1, 2, \cdots, m_2 - 2$) will progress to V_{i+1}; and individuals in the V_{m_2-1} class will proceed to the susceptible class. Those whose age post recent recoverey is m_1 will also move to susceptible class. Individuals in the I class will move to $r(nT^-, 0)$ as recent recovery, and those in V_v and R_v enter the class V_1, as their age since the most recent vaccination is T. Thus, prior to

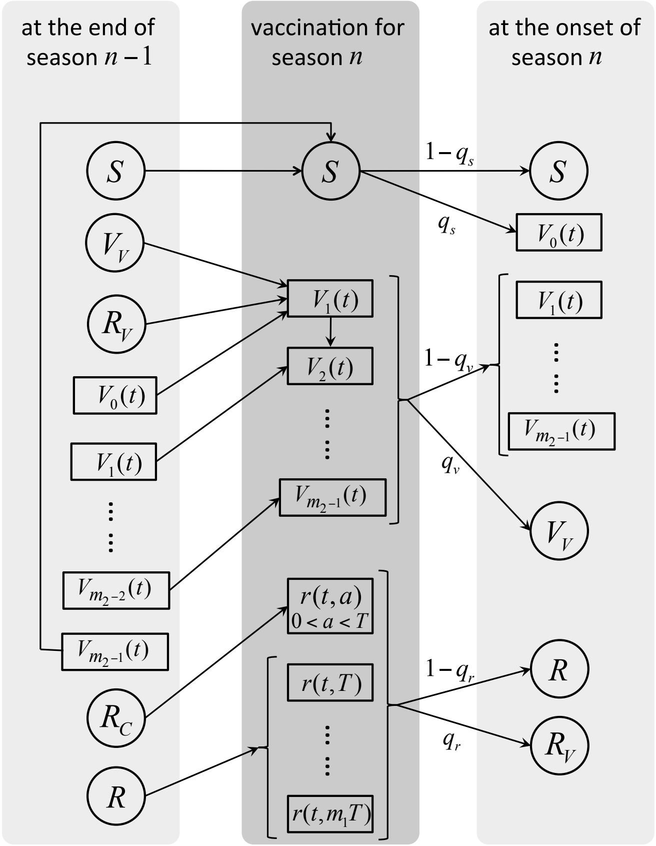

Figure 2: Model diagram for transitions and compartments between seasonal epidemics.

vaccination for the upcoming $n+1$ season, we have the following changes for each compartment:

$$
\begin{aligned}
S(nT) =& S(nT^-) + V_{m_2-1}(nT^-), \\
V_0(nT) =& 0, \\
V_1(nT) =& V_0(nT^-) + V_v(nT^-) + R_v(nT^-), \\
V_{i+1}(nT) =& V_i(nT^-), \quad i = 1, 2, ..., m_2 - 2, \\
V_v(nT) =& 0, \\
R_v(nT) =& 0, \\
I(nT) =& 0, \\
r(nT, a) =& r(nT^-, a), \\
r(nT^-, 0) =& I(nT^-).
\end{aligned}
\tag{2.1}
$$

We divided the total population into three classes by the following rules:

1) Susceptible individuals ($\mathbf{S}(nT)$): without having any cross-protection effects of natural infection or vaccination;

2) Previously vaccinated individuals ($\mathbf{V}(nT)$): having some level of cross-protection induced by vaccination; and

3) Recovered individuals ($\mathbf{R}(nT)$): having some level of cross-protection induced by natural infection.

We now include the distribution of vaccines into the model. Suppose N is the total population size, and there is μN vaccine doses available, where $0 < \mu < 1$. We distribute vaccines to the three classes such that $q_s\mathbf{S}(nT) + q_v\mathbf{V}(nT) + q_r\mathbf{R}(nT) = \mu N$, where q_s, q_v, and q_r represent the proportions of susceptible, previously vaccinated, and previously recovered individuals, respectively, who receive vaccines for the upcoming season. Within each class, we assume vaccines are evenly distributed regardless of age structure post vaccination or infection. After vaccination, we consider the population dynamics as follows:

1) Newly vaccinated individuals from **S**, **V** and **R** will move to the compartments V_0, V_v and R_v, respectively;

2) There is no difference in the level of immunity for the newly vaccinated individuals regardless of their history of vaccination or infection within the V_v or R_v classes; and

3) The effect of vaccine-induced protection on disease transmissibility will be $\beta_v^0 \geq \bar{\beta}_v \geq \bar{\beta}_r$, where β_v^0, $\bar{\beta}_v$, and $\bar{\beta}_r$ represent the baseline transmission rates of newly vaccinated individuals who were susceptible, previously vaccinated, or previously recovered, respectively.

Hence, we have the following initial conditions for the upcoming season:

$$
\begin{aligned}
S(nT^{+}) =&(1-q_s)S(nT),\\
V_0(nT^{+}) =&q_s S(nT),\\
V_i(nT^{+}) =&(1-q_v)V_i(nT), \quad i=1,2,...,m_2-1,\\
V_v(nT^{+}) =&q_v \sum_{i=1}^{m_2-1} V_i(nT),\\
R_v(nT^{+}) =&q_r \int_0^{m_1 T} r(nT,a)\,\mathrm{d}a,\\
r(nT^{+},a) =&(1-q_r)r(nT,a),
\end{aligned}
\tag{2.2}
$$

In order to introduce infection into the population, we move one individual from the susceptible class $S(nT^{+})$, or recovered class $r(nT^{+},a)$, or vaccinated class $V_i(nT^{+})$ to the infection class $I(nT^{+})$. Therefore, we can rewrite the initial conditions for the corresponding classes as:

$$
\begin{aligned}
S(nT^{+}) =&\max\{(1-q_s)S(nT)-1,0\},\\
I(nT^{+}) =&\max\{\,S(nT^{+})-(1-q_s)S(nT),0\}.
\end{aligned}
\tag{2.3}
$$

or

$$
\begin{aligned}
\max_a\{r(nT^{+},a)\} =&\max\{\max_a\{(1-q_r)r(nT,a)-1\},0\},\\
I(nT^{+}) =&\max\{\,\max_a\{r(nT^{+},a)\}-\max_a\{(1-q_r)r(nT,a)\},0\}.
\end{aligned}
\tag{2.4}
$$

or

$$
\begin{aligned}
\max_i\{V_i(nT^{+})\} =&\max\{\max_i\{(1-q_v)V_i(nT)-1\},0\},\\
I(nT^{+}) =&\max\{\,\max_i\{V_i(nT^{+})\}-\max_i\{(1-q_v)V_i(nT)\},0\},
\end{aligned}
\tag{2.5}
$$

where $nT(nT^{-})$ is a point in time after season n has ended (before the start of vaccination for

season $n+1$). In summary, we can define the initial conditions for season n as

$$
\begin{aligned}
S^0 =& S(nT^+);\\
V_i^0 =& V_i(nT^+), \quad i = 0, 1, ..., m_2 - 1;\\
V_v^0 =& V_v(nT^+),\\
R_v^0 =& R_v(nT^+),\\
\Phi(a) =& r(nT^+, a).
\end{aligned}
\tag{2.6}
$$

where nT^+ represents a point in time after vaccination for season $n+1$, and before the onset of epidemic in season $n+1$.

3 Full model

Combining the dynamics of within- and between-seasons of the model with the initial conditions described above for each season, we can express $R(t)$ from equation (1.1), using the characteristic method, by

$$
\begin{aligned}
R(t) &= \int_0^{m_1 T} r(t,\, a)\,\mathrm{d}a,\\
&= \int_0^{t-nT} \gamma I(t-a) e^{-\int_0^a \kappa_r(t+u-a,u) I(t+u-a)\,\mathrm{d}u}\,\mathrm{d}a\\
&\quad + \int_{t-nT}^{m_1 T} \Phi(a-(t-nT)) e^{-\int_0^{t-nT} \kappa_r(u,a-(t-nT)+u) I(u)\,\mathrm{d}u}\,\mathrm{d}a,\\
&= \int_0^{t-nT} \gamma I(t-a)\,\mathrm{d}a \qquad\qquad (\kappa_r(t,\, a) = 0 \text{ for } a \le t-nT)\\
&\quad + \int_{t-nT}^{m_1 T} \Phi(a-(t-nT)) e^{-\int_0^{t-nT} \kappa_r(u,a-(t-nT)+u) I(u)\,\mathrm{d}u}\,\mathrm{d}a
\end{aligned}
$$

where $t \in [nT,\ (n+1)T)$. Therefore, we have the full model in the form:

$$
\begin{aligned}
\frac{\mathrm{d}S(t)}{\mathrm{d}t} =& -\kappa_s(t)I(t)S(t) + \Phi(m_1T-(t-nT))e^{-\int_0^{t-nT}\kappa_r(u,m_1T-(t-nT)+u)I(u)\,\mathrm{d}u},\\
\frac{\mathrm{d}V_i(t)}{\mathrm{d}t} =& -\kappa_v^i(t)I(t)V_i(t), \qquad\qquad i=0,1,...,m_2-1,\\
\frac{\mathrm{d}V_v(t)}{\mathrm{d}t} =& -\bar{\kappa}_v(t)I(t)V_v(t),\\
\frac{\mathrm{d}R_v(t)}{\mathrm{d}t} =& -\bar{\kappa}_r(t)I(t)R_v(t),\\
\frac{\mathrm{d}I(t)}{\mathrm{d}t} =& -\gamma I(t) + \kappa_s(t)I(t)S(t)\\
&+\bar{\kappa}_v(t)I(t)V_v(t) + \sum_{i=0}^{m_2-1}\kappa_v^i(t)I(t)V_i(t)\\
&+\bar{\kappa}_r(t)I(t)R_v(t)\\
&+\int_{t-nT}^{m_1T}\kappa(t,a)I(t)\Phi(a-(t-nT))e^{-\int_0^{t-nT}\kappa_r(u,a-(t-nT)+u)I(u)\,\mathrm{d}u}\,\mathrm{d}a,
\end{aligned}
\tag{3.1}
$$

where $t \in [nT,\ (n+1)T)$; $\bar{\kappa}_v(t) = \bar{\beta}_v P_v(t)$ and $\bar{\kappa}_r(t) = \bar{\beta}_r P_r(t)$ represent transmission rates for newly vaccinated individuals who received vaccines within the past m_2 seasons, and individuals who had recovered from infection within the past m_1 seasons, respectively; with the baseline values $\bar{\beta}_v$ and $\bar{\beta}_r$ corresponding to seasonality factors $P_v(t)$ and $P_r(t)$, respectively. The Volterra intergro-differntial quation system (3.1) satisfies the hypotheses stated by Miller ([7], P338) that are sufficient to ensure the existence, uniqueness, and continuity of solutions on $t \in [nT, (n+1)T)$. Furthermore, for any non-negative initial conditions,

$$S(0) > 0, \quad V_i(0) \geq 0, \quad V_v \geq 0, \quad R_v \geq 0, \quad I(0) \geq 0,$$

$$r(0,a) \geq 0, \quad r(t,0) \geq 0, \quad i = 0,2,..,m_2-1, \quad a,t \in R/R^-,$$

system (3.1) (with $r(t,a)$ replaced by (3.2)) has a unique solution. By using the variation-of-constant formula to individual equations, one can verify the non-negativity of the equations. This proves the well-posedness of the full model.

One can also show the well-posedness of the partial differential equation for $r(t, a)$. Fixing (t, a) and letting $W(s) = r(t + s, a + s)$, one can replace the equation for $r(t, a)$ in (3.1) by an ordinary differential equation,

$$\frac{\mathrm{d}W(s)}{\mathrm{d}s} = B(s)W(s), \tag{3.2}$$

where $B(s) = -\kappa_r(t + s, a + s)I(t + s)$ with the initial condition $W(0) = r(t, a)$. We can show the non-negativity of $I(t)$ by solving its related equation; therefore, $W(s) \geq 0$ and its uniqueness can be easily obtained.

4 Computer implementation

We implemented the model using C++ and Matlab software package to perform simulations of the model. The integro-differential equations were solved numerically using forward-time central-space algorithm and midpoint method. We simulated several scenarios in the presence and absence of seasonal vaccination (represented in Figure 3-8 in main text). For each scenario, we considered a combination of m_1 and m_2, and ran 100 independent simulations for 12 seasons. The algorithm for simulations proceeded with the following steps:

Step 1: At the beginning of each season, we assigned initial values for (S_0, V_0, R_0) to different population compartments. For the first season of the simulation run, we set the same distribution in each scenario for different m_1 and m_2. For subsequent seasons, we chose the population distribution simulated from previous season ($(S(nT^-), V(nT^-), R(nT^-)),\ n = 1, 2, ..., 11$);

Step 2: Based on the chosen distribution, the feasible region of $(q_s,\ q_v)$ is determined as $\Omega = \{(q_s,\ q_v)|0 \leq q_s, q_v \leq 1, q_sS^0 + q_vV^0 \leq \mu N\}$. We discretized the space $\left[0, \frac{\mu N}{S^0}\right] \times \left[0, \frac{\mu N}{V^0}\right]$, $S^0 > 0, V^0 > 0$ on a square grid (with a side-length grid of 0.02), and used each pair of $(q_s,\ q_v)$ on the node of the grid to regroup the total population into $m_2 + 5$ subpopulations,

$$(S^0, V_0^0, V_1^0, ..., V_{m_2-1}^0, V_v^0, R_v^0, \Phi, I),$$

with (2.2)− (2.6) as the initial conditions for simulations of the current season.

Step 3: We numerically solved the model governed by the integral-differential equations system to determine the dynamics of epidemic within a season, for all possible pairs of $(q_s,\, q_v)$, and stored the values of all population compartments at the end of the season. Iterations were ended when $I(t) < \epsilon_I$ (a given small threshold) or $t \geq T$. The remaining subpopulation $I(t)$ was moved to $r(0,t)$, and $I(t)$ class was set to zero.

Step 4: Two strategies were examined individually to proceed with the selection of the population distribution (S_0, V_0, R_0) for each subsequent season:

4.1. *Random selection*: a pair of $(q_s,\, q_v)$ was randomly selected in space Ω regardless of outputs at the end of each season, and the simulated population distribution

$$(S(nT), V_0(nT), V_1(nT), ..., V_{m_2-1}(nT), V_v(nT), R_v(nT), r(a,(nT)))$$

was used to compute (S_0, V_0, R_0) according to the rule mathematically presented in (2.1).

4.2. *Optimal selection*: we computed the final size of epidemic (i.e., the total number of infections throughout the epidemic), given all possible pairs of $(q_s,\, q_v)$:

$$J(q_s,\, q_v) = \int_{nT}^{(n+1)T} \gamma I(\eta)\, \mathrm{d}\eta,$$

for season n, $n = 1, 2, ..., 12$. The pair $(q_s,\, q_v)$ that gave

$$J^n_{min} = \min_{(q_s,\, q_v)\in\Omega} J(q_s,\, q_v),$$

was identified, and the associated simulation results were adopted to compute (S_0, V_0, R_0) according to (2.1). If there were more that one pair satisfying the minimum epidemic final size, we chose one randomly. This highlights our observations that the optimal vaccine distribution may not be uniquely determined.

We repeated steps 1-4 for all 12 simulated seasons in each scenario.

Remark 4.1 In each scenario of $(q_s,\, q_v)$, the fraction of previously recovered individuals (q_r) who are vaccinated for the current season was determined using the relation $q_sS + q_vV + q_rR = \mu N$. In simulation plots, under the circumstances that a pair of $(q_s,\, q_v)$ on grid satisfied $q_sS + q_vV > \mu N$, we compared two cases of maximum vaccine distribution: 1) all vaccines are given to the S subpopulation; 2) all vaccines are given to the V subpopulation. The resulting simulation outputs of the pair $(q_s,\, q_v)$ with the lower epidemic final size was adopted.

References

[1] Couch RB, Kasel JA, Immunity to influenza in man. Annu Rev Microbiol 1983; 37: 529-49.

[2] Diekmann O, Heesterbeek J.A.P, Mathematical Epidemiology of Infectious Diseases, Chichester: Wiley, 2000.

[3] Metz JAJ, Diekmann O, The dynamics of physiologically structured populations. Springer, New York 1986.

[4] d'Onofrio A, On pulse vaccination strategy in the SIR epidemic model with vertical transmission, Appl. Math Lett 2005; 18: 729 - 732.

[5] Dushoff J, Plotkin JB, Levin SA, Earn DJ, Dynamical resonance can account for seasonality of influenza epidemics, Proc Natl Acad Sci U S A. 2004; 101: 16915-16916.

[6] Shulgin B, Stone J, Agur A, Pulse vaccination strategy in the SIR epidemic model, Bull. Math. Biol. 1998; 60: 1123 - 1148.

[7] Miller RK, Nonlinear Volterra Integral Equations, Benjamin, Menlo Park, California,1971.
